# Long-term trends in supply and sustainability of the health workforce in remote Aboriginal communities in the Northern Territory of Australia

**DOI:** 10.1186/s12913-017-2803-1

**Published:** 2017-12-19

**Authors:** Yuejen Zhao, Deborah J. Russell, Steven Guthridge, Mark Ramjan, Michael P. Jones, John S. Humphreys, Timothy A. Carey, John Wakerman

**Affiliations:** 1 Menzies School of Health Research, PO Box 41096, Casuarina, NT 0811 Australia; 2 Monash Rural Health, Monash University, PO Box 666, Bendigo, VIC 3552 Australia; 3 Faculty of Human Sciences, Macquarie University, North Ryde, NSW 2109 Australia; 4 Centre for Remote Health, Flinders University and Charles Darwin University, PO Box 4066, Alice Springs, NT 0871 Australia; 5 Flinders Northern Territory, College of Medicine & Public Health, Flinders University, PO Box U362, Casuarina, NT 0815 Australia

**Keywords:** Remote health, Remote workforce, Rural workforce, Health workforce, Fly-in/fly-out, Rural health services, Aboriginal, Aboriginal health practitioner, Remote area nurse, Workforce supply

## Abstract

**Background:**

International evidence suggests that a key to improving health and attaining more equitable health outcomes for disadvantaged populations is a health system with a strong primary care sector. Longstanding problems with health workforce supply and turnover in remote Aboriginal communities in the Northern Territory (NT), Australia, jeopardise primary care delivery and the effort to overcome the substantial gaps in health outcomes for this population. This research describes temporal changes in workforce supply in government-operated clinics in remote NT communities through a period in which there has been a substantial increase in health funding.

**Methods:**

Descriptive and Markov-switching dynamic regression analysis of NT Government Department of Health payroll and financial data for the resident health workforce in 54 remote clinics, 2004–2015. The workforce included registered Remote Area Nurses and Midwives (nurses), Aboriginal Health Practitioners (AHPs) and staff in administrative and logistic roles. Main outcome measures: total number of unique employees per year; average annual headcounts; average full-time equivalent (FTE) positions; agency employed nurse FTE estimates; high and low supply state estimates.

**Results:**

Overall increases in workforce supply occurred between 2004 and 2015, especially for administrative and logistic positions. Supply of nurses and AHPs increased from an average 2.6 to 3.2 FTE per clinic, although supply of AHPs has declined since 2010. Each year almost twice as many individual NT government-employed nurses or AHPs are required for each FTE position.

Following funding increases, some clinics doubled their nursing and AHP workforce and achieved relative stability in supply. However, most clinics increased staffing to a much smaller extent or not at all, typically experiencing a “fading” of supply following an initial increase associated with greater funding, and frequently cycling periods of higher and lower staffing levels.

**Conclusions:**

Overall increases in workforce supply in remote NT communities between 2004 and 2015 have been affected by continuing very high turnover of nurses and AHPs, and compounded by recent declines in AHP supply. Despite substantial increases in resourcing, an imperative remains to implement more robust health service models which better support the supply and retention of resident health staff.

## Background

International evidence suggests that a key to improving health and attaining more equitable health outcomes for disadvantaged populations is a health system underpinned by strong primary care which is responsive to health-related needs [1, 2]. For primary care to function effectively it is critical that sufficient suitably-skilled and culturally-appropriate primary care practitioners are located where populations can gain timely access to services [3]. Across the globe governments face a perpetual struggle to ensure adequate and accessible primary care services for populations living in rural and remote locations.

Aboriginal and Torres Strait Islander Australians (hereafter referred to collectively as Aboriginal Australians), like indigenous populations in many other countries, experience poorer health than the general population – exemplified by a life expectancy at birth that is 10 years less than non-Aboriginal Australians [4]. In Australia, these poorer health outcomes reflect significant associations between geographical remoteness and population health, which is related to socioeconomic disadvantage, higher levels of disease risk factors, reduced accessibility to health services, and higher exposure to a range of other environmental risks [5]. Nowhere is this disadvantage more acutely evident than in remote Aboriginal communities, where ensuring an adequate supply of primary care practitioners remains problematic [6, 7].

The Northern Territory (NT) of Australia, which covers approximately 1.3 million km^2^, is sparsely settled, with a population of less than 250,000 people, 30% of whom identify as Aboriginal Australians. Most (81%) Aboriginal Territorians live in remote communities [8]. Primary care delivery in these communities is heavily reliant on resident Remote Area Nurses and Midwives (nurses) and Aboriginal Health Practitioners (AHPs), with professional support provided by telehealth, and scheduled intermittent visits from medical and allied health practitioners. An inadequate health workforce supply has long been recognised as a key factor which limits primary care service provision in this setting. Issues highlighted in relation to current and future NT health workforce include difficulties in attracting and developing a suitably skilled workforce and the high mobility of medical practitioners and nurses. Each of these issues can have a negative impact on both the recruitment and retention of Aboriginal community health staff and patient continuity of care [7, 9, 10].

As a consequence of the high mobility of resident nurses there is heavy reliance on short-term agency and casual nurses [9, 11]. The NT government, in an attempt to provide a career pathway in to nursing practice in remote Aboriginal primary health care environments, offers a *‘Transition to Primary Health Care Practice’* program of study and training in partnership with a local university [12]. In this program, nurses at level 3 (N3) are provided with the minimum skills and knowledge needed for independent practice in remote Aboriginal primary health care. The degree to which the supply of nurses in remote Aboriginal communities is supported by the development of these N3 clinical nurses, however, is not known.

There have also been specific concerns about the ongoing supply of NT AHPs, with evidence indicating that not only has the number of registered NT AHPs between 1999 and 2010 declined, but this has been reflected in difficulties filling vacant AHP positions [13, 14]. Underlying barriers to attracting and training increased numbers of AHPs include: increased numeracy and literacy requirements; periods of training away from the community in order to attain minimum AHP qualifications; relatively poor pay and career opportunities; and high community demands and professional expectations [14]. However, the extent to which barriers to AHP practice are reflected by changes in AHP supply over time is not well documented.

Within the last decade, significant investments have been made in the NT primary care health workforce servicing remote Aboriginal communities. In mid-2007 the *NT Emergency Response* (NTER) was initiated, followed in 2008 by the *Expanding Health Service Delivery Initiative*, and in 2012 by the *Stronger Futures Northern Territory* initiative [7, 15, 16]. These programs resulted in increased funding for general government services, including: funds committed to improving housing and primary care infrastructure to support staff recruitment and retention; and funds for employing additional primary care staff. These positions included additional community-based nurses providing health care in a single community, as well as new roles such as: non-community-based continuing quality improvement facilitators; child health coordinators; chronic disease coordinators; public health coordinators; and area managers. People in these roles provide support across multiple communities. Little is known, however, about the impact that these investments have had on primary care practitioner supply and the sustainability of that supply in remote Aboriginal communities. There remains a dearth of literature quantifying primary care practitioner supply in remote NT Aboriginal communities, how supply has changed over time, and the significance of associations with policy implementation and a range of other factors such as workforce demographics and community characteristics. Further, a lack of reliable information hampers planning for the future NT health workforce [6].

Bearing in mind these important gaps in our knowledge, the aim of this paper is to describe temporal changes in primary care practitioner supply in NT remote health clinics between 2004 and 2015. More specifically, the paper will describe temporal changes in workforce supply: in all NT government remote health clinics; according to key demographic and community characteristics; and at a health clinic level.

## Methods

This research is part of a larger study assessing the impact and cost of short-term health staff in NT remote clinics, which is described elsewhere [17]. This analysis uses data from NT Government Department of Health (NTG DOH) Personnel Information and Payroll System (PIPS) and Government Accounting System (GAS) datasets. PIPS data captured all staff directly employed by the NTG DOH in 54 NTG-governed remote health clinics at any time between September 2003 and December 2015 inclusive. These data include “agency” nurses recruited through nurse employment agencies and employed by NTG DOH on temporary or casual contracts. The payroll data were available for each fortnightly pay period.

NT GAS financial data captured nurses employed directly by a nursing agency to work in any of the 54 NTG DOH remote health clinics at any time between July 2003 and June 2016. GAS expenditure on agency nurse labour hire costs were used to estimate how many aggregated full-time equivalent (FTE) agency employed nurses were working in remote health services. The estimate used the standard NTG DOH formula of agency labour hire costs divided by twice the Departmental annual average nurse personnel cost.

Four metrics were used to assess variations in supply of community-based staff over time:

1. Total number of unique employees (sum of individuals employed in each year);
2. Average annual headcounts (average number of individuals employed in each pay period in each year);
3. Average FTE (average FTE employed in each pay period in each year); and
4. Agency employed nurse FTE estimates.

Using these four different supply metrics provides useful information that is lost if a single metric is used in isolation. Comparing average headcounts (measure 2) with average FTE (measure 3) provides information about the extent to which employees work part-time. Similarly, comparing unique employees (measure 1) with average headcounts (measure 2) provides information about employee turnover. The ratio of unique individual employees to Average FTE was used to measure the number of unique persons required per FTE annually.

Categorical variables measuring demographic characteristics of community-based primary care employees were as follows:

- Employment category: nurse; AHP; and, ‘other’(administrative officers, logistic support (drivers, cleaners and gardeners), technical, medical, allied health, and trainees);
- Employment level: AHP levels 1, 2 or 3, and 4; nurse levels 3, 4, and 5–7, whereby lower levels are more junior and higher levels more senior;
- Gender: male, female; and
- Age categories: <30 years, 30- < 50, ≥50.

Categorical variables measuring community characteristics were as follows:

- Community population type and size: predominantly non-Aboriginal residents; predominantly Aboriginal residents and very small (<200 residents), small (200–349 residents), medium-sized (350–799 residents), and large (≥800 residents); and
- Remoteness according to the distance to the major regional centres of Darwin or Alice Springs (whichever was closer): <200 km; 200–299 km; and ≥300 km.

Community population size was extracted from 2014 electronic primary care patient records in which an indicator for the current catchment population for each clinic is actively managed by clinic staff. Distances to Darwin or Alice Springs were measured using Google Maps straight line distance in kilometres.

In addition, two-state Markov-switching dynamic regression models were used to investigate supply sustainability of FTE nurses and AHPs at each health clinic and on average for all health clinics. The average supply was calculated by summing nurse and AHP FTE for all clinics in each pay period and dividing by 54 (the number of clinics included in the analysis). This analytic approach enabled two states to be identified: a high and a low supply state for each clinic. It was hypothesised that the effect of substantial boosts in government funding during the study period would be associated with increased nurse and AHP supply. The use of dynamic models allowed for a rapid adjustment in supply from one pay period to the next.

Ethics approval was received from the Human Research Ethics Committee of the NTG DOH and Menzies School of Health Research (2015–2363).

## Results

### Total workforce supply of community-based staff over time

**Fig. 1:**
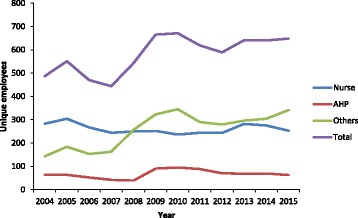
Total number of unique employees by employment category and time, 2004–2015, Northern Territory

Total workforce supply, as measured by numbers of unique employees, increased in the 12-year study period, although the increase was not constant (Fig. 1). A rapid (50%) increase in overall employee numbers was evident from 2007 to 2009, largely for ‘other’ employees. For AHPs a decline in the number of unique individuals occurred between 2004 and 2008, followed by a 150% increase from 2008 to 2010. Following 2010 the number of unique individual AHPs again declined to the levels recorded in 2004. The increases in workforce supply coincided with the initiation and early phases of the NTER which commenced in 2007. Nurse supply, when measured by unique individuals, showed little change over the study period.

**Fig. 2:**
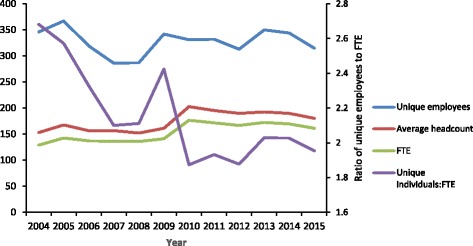
Comparison of annual unique persons, average annual headcount and FTE, nurses and Aboriginal Health Practitioners, 2004–2015, Northern Territory

Figure 2 presents patterns of nurse and AHP supply for the study period and demonstrates that between 2009 and 2010 there was growth in both average headcounts and FTE (Fig. 2). Figure 2 also demonstrates a substantial difference in supply if measured by unique employees and average headcounts, whereas average headcount and FTE measures closely parallel each other throughout the study period. The ratios of unique employees to FTE were higher in 2004, 2005, and 2009 with levels around 2.4–2.6, and lower in later years with levels of approximately 1.8–2.0.

**Fig. 3:**
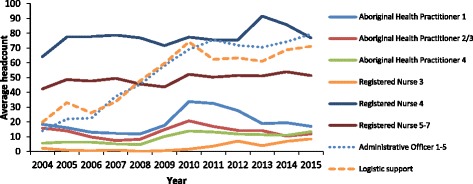
Average headcount of remote clinic employees by employment category and level, 2004–2015, Northern Territory

Analysis of workforce supply over time, measured by average headcount in different employment categories and employment levels (Fig. 3), demonstrates that the largest increases in workforce supply in remote NT clinics were in the logistic support and administrative categories. These supply increases occurred from 2004 to 2010. Increases in headcounts of AHPs occurred across all levels from 2008 to 2010, after which there was a decline in the number of levels 1 and 2/3. Small increases in nursing headcounts (at levels 3, 4, and 5–7) occurred from 2009 to 2010 and continued for level 3 s until 2015, whereas supply of level 5–7 s plateaued from 2010.

**Fig. 4:**
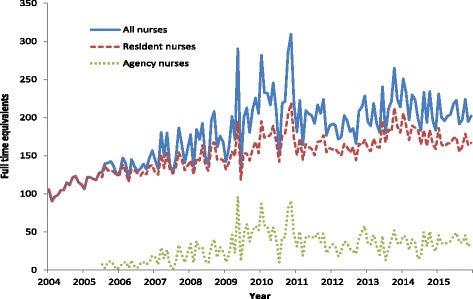
Trends in full-time equivalent agency and NT DOH employed nurses, 2004–2015, Northern Territory

Remote clinic supply of agency employed FTE nurses, while showing considerable variation from month to month, tended to increase in parallel with NT DOH employed nurses between 2005 and 2011, thereafter plateauing at a level of approximately 30 to 40 FTE, or about 15% to 20% of the total nurse workforce (Fig. 4).

### Total workforce supply of community-based staff over time, by key demographic and other characteristics

Table 1 presents key characteristics of workforce supply before and after the increase in funding associated with the NTER. The increased funding was associated with a statistically significant increase in AHPs (*p* < .01) but not of nurses. Additionally, there was a statistically significant shift in the age of the nursing workforce, with an increase in average headcount of those nurses aged 50 years and older (*p* < .01), from 48.3 to 78.2. This was equally driven by recruitment of older nurses and ageing of the nurse workforce. Reductions in the average headcount of nurses occurred in the two younger age groups. Workforce age composition changes were different for the AHP workforce supply, which had non-significant increases in average headcounts for those aged under 30 years and 50 years and over, but a significant increase in the average headcount for AHPs in the 30 to 49-year category (*p* < .01), which almost doubled from 19.2 to 35.3.

**Table 1:** Average headcount by employment group, before and after NT Emergency Response initial funding flow

	Nurse		AHP		Total	
	Pre^	Post^^	Pre^	Post^^	Pre^	Post^^
Total	120.8	135.1	31.6	**52.2	152.4	187.3
Age < 30	8.1	6.3	2.2	2.8	10.3	9.0
Age 30–49	64.5	50.7	19.2	**35.3	83.7	85.9
Age 50+	48.3	**78.2	10.1	14.1	58.5	**92.3
Female^†^	97.7	103.4	21.2	**40.4	118.9	143.8
Male	29.0	31.8	5.0	**11.7	34.0	43.5
<200 km	22.2	25.4	18.0	21.7	40.3	47.1
200-299 km	27.3	32.9	1.9	**7.8	29.2	*40.7
≥300 km	52.6	58.2	11.5	**21.1	64.0	79.3
non-Aboriginal	18.7	18.6	0.2	**1.6	18.9	20.2
population < 200	7.8	11.2	2.9	*7.0	10.7	*18.2
population 200–349	9.7	12.9	3.0	3.5	12.7	16.4
population 350–799	28.8	30.8	9.5	**19.7	38.2	*50.5
population > 800	55.8	61.6	16.0	20.4	71.8	82.0

^†^Age and gender data averaged for second half of 2007 and 2008 (age and gender data not available prior to 13th pay period in 2007); ^2004–2008 Pre NTER funding; ^^2009–2015 post NTER initial funding boost; *p < .05; **p < .01

Supply of both male and female AHPs increased significantly (*p* < .01) following the NTER while there was a non-significant change in the supply of male and female nurses. Following the NTER, increases in both the percentage and the number of AHPs was significantly greater in communities further than 200 km from Darwin or Alice Springs (*p* < .01), whereas the percentage increase in nurse supply was non-significant in all categories of distance from major regional centres. Between the two periods, health services in those communities with predominantly non-Aboriginal populations experienced little change in combined nurse and AHP supply, although there was a statistically significant increase in the small number of AHPs (*p* < .01). Following the NTER, the smallest remote communities (population < 200) experienced significant increases in AHP and total (nurse and AHP) (*p* < .05) supply, though increases in nurse supply alone were non-significant. Changes in workforce supply for the largest remote communities were non-significant.

### Workforce supply of community-based staff at a health clinic level over time

**Fig. 5:**
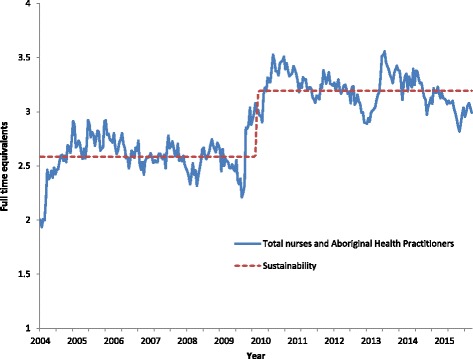
Average FTE workforce supply of nurses and AHPs per clinic, 2004–2015, Northern Territory

Average FTE workforce supply of nurses and AHPs for all clinics combined is presented in Fig. 5. The results demonstrate fluctuations from pay period to pay period and substantial increases in supply at the time of increased funding around 2010 and 2013. The increased resourcing was associated with an average clinic FTE increase from approximately 2.6 to approximately 3.2 nurses and AHPs. The sustainability model produced by the two-state Markov chain modelling process demonstrates a sustained increase in supply associated with increased funding. A further feature evident in Fig. 5 from a visual inspection of FTE changes over time is an apparent deterioration or “fading” of supply after the initial increase associated with each period of increased funding.

**Fig. 6:**
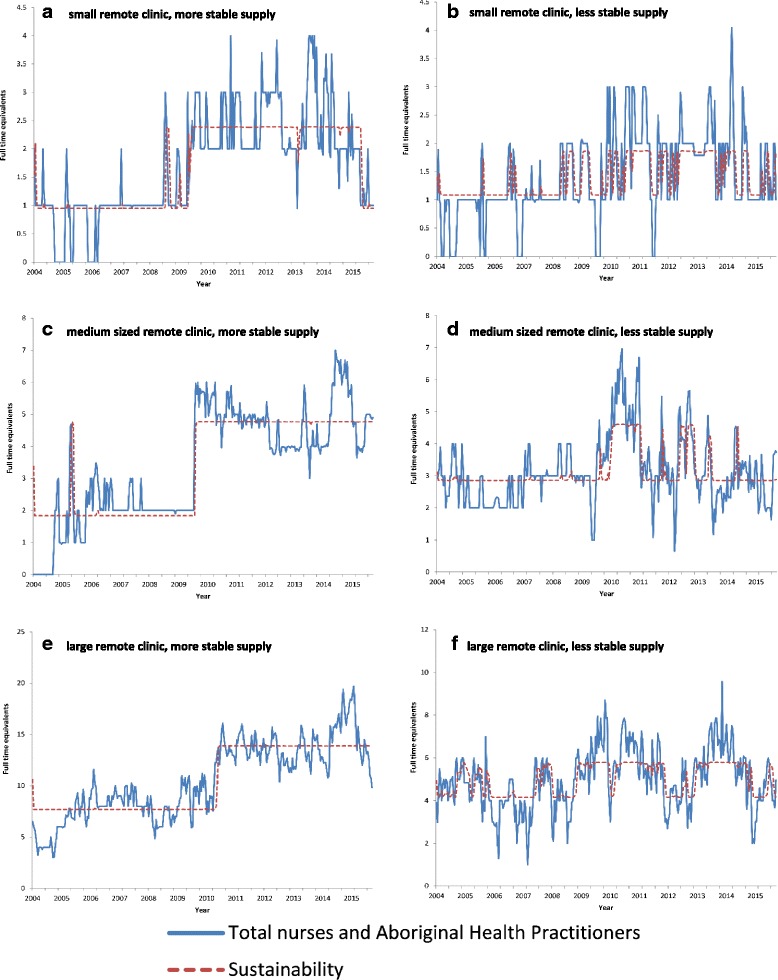
**a**-**f** Exemplar nurse and AHP FTE supply in remote community clinics

The workforce supply was also assessed at each of the 54 NTG DOH remote health clinics. Six example clinics are presented as Fig. 6a to 6f. Two clinics are drawn from small communities, two from medium-sized communities, and two from large communities. For each community size, a clinic with a stable workforce supply and a clinic with a less stable workforce supply have been selected.

Figure 6a and b provide the data from clinics in two small communities. The clinic in 6a demonstrates a sustained increased in nurse and AHP staffing from approximately 2009, though with evidence of recent deterioration. In 2005 and 2006 this community frequently had no NTG-employed nurses or AHPs, whereas in recent years the clinic mostly had at least 2, often 3, and sometimes 4 nurses and AHPs. The clinic in Fig. 6b demonstrates a less sustained pattern of nurse and AHP supply. In this community FTE supply frequently shifts between 1 and 2 nurses and AHPs with only two longer periods when the clinic consistently had 2 or more nurses and AHPs.

Figure 6c and d provide the data from clinics in two medium sized communities. The clinic in 6c demonstrates a relatively sustained supply of nurse and AHP FTE staff, characterised by a sharp increase in supply from approximately 2.0 FTE before 2009 to almost 5.0 FTE after 2009. The workforce for this clinic is, however, exceptional. The majority of clinics in medium sized Aboriginal communities had workforce supply patterns more similar to the clinic modelled in Fig. 6d. Staffing of the remote clinic shown in Fig. 6d experienced a boost in nurses and AHP FTE supply at a similar time to that of the clinic graphed in Fig. 6c, however the increase has not been sustained. After a period between 2010 and 2011 of a larger workforce supply, there have been subsequent periods of many fewer staff with times when staffing fell to one FTE NTG DOH nurse/AHP and most periods fluctuating around 3.0 FTE NTG DOH employed nurses and AHPs.

Figure 6e and f provide data from clinics in two large communities. The health workforce supply modelled in Fig. 6e again demonstrates the marked increase in NTG DOH employed nurse and AHPs supply that occurred related to the NTER. The community transitioned from an average supply of approximately 7.5 FTE nurses and AHPs prior to the NTER, to a level almost double that, at approximately 14.0 FTE. This later level has generally been sustained. In contrast, in the clinic shown in Fig. 6f, nurse and AHP supply switches between a supply of approximately 4.0 FTE nurses and AHPs to approximately 6.0 FTE, though mostly with longer periods at the higher supply level after 2009 compared to before 2009.

After an initial increase in supply, associated with increased funding, fading of supply is evident in the medium and large clinics that we selected to model, and in many of the 54 clinics examined. The graphs presented for individual clinics also demonstrate a second pattern, of cycles of higher and lower staffing levels. The period of the cycles varies between clinics and at different times. In the clinic shown in Fig. 6d, in a medium-sized community, increased supply is sustained for approximately one year at levels above 4.5 FTE before falling to 3.0 FTE, and then briefly recovers. For the clinic at Fig. 6f there are recurring periods of one to two years in which there is a larger workforce supply followed by a period of relative instability and reduced supply.

## Discussion

Substantial changes in primary care practitioner supply have occurred in remote NT communities between 2004 and 2015. Analysis reveals overall increases in supply over this period, especially for employees providing administrative and logistic support. Improved levels of administrative and logistic support are likely to allow nursing and AHP clinicians to focus more time on clinical services. A further benefit may be that increased employment of local staff provides opportunity for better retention of organisational knowledge and improved community engagement in a setting of very high nurse turnover. Research on the provision of dental services in a Queensland Aboriginal and Torres Strait Islander community has reported that continuity of permanently employed dental assistants helped counteract the negative impact of the turnover of dentists [18].

Growth in overall health workforce supply did not occur at a steady rate. Among NTG employed nurses and AHPs, two bursts in increased FTE supply were evident – one during 2009–2011 and another during 2013–2015, each coinciding with the release of additional funds. On average, the extra resourcing manifested as a 23% increase, from 2.6 to about 3.2 FTE nurses and AHPs, for individual remote health clinics. The effects on nurse and AHP supply in individual clinics, were, however, far from uniform during the study period. There was also evidence that after the funding increases there was a tendency for workforce FTE supply to fade over time, reflecting the difficulties in recruitment and retention of nurses and AHPs, and in sustaining primary care services in remote areas. This evidence suggests that while increased funding is likely to be part of the solution to difficulties in attracting and developing an appropriately skilled primary health care workforce in remote communities, clearly other strategies are also needed. These may relate, for example, to training and preparation to work effectively in a remote setting, or governance issues, or levels of management and clinical support, or overall critical mass – that is, a substantially larger resident workforce is required to deal with the high health need.

Our study identified some exemplary communities, which were able to double their nursing and AHP workforce at the time of the increased funding, and continued to achieve relative stability in supply over time through to 2015. It is possible that by studying communities where these issues are being addressed satisfactorily we will learn what else needs to be done in addition to funding increases in order to rectify workforce problems in remote Australia and make an important contribution to reducing the disparity in health outcomes for the people who live there.

Most communities, however, were only able to increase their NTG employed nursing and AHP workforce to a much smaller extent or not at all, and experienced ongoing workforce instability. These communities cycled at varying rates between periods of relatively higher supply of nurses and AHPs and periods of lower supply, a fragility in supply which may compromise the consistent provision of high quality care for remote Aboriginal populations. These findings are corroborated by the high ratios of unique employees to FTE which suggest very high levels of turnover, with 1.8–2.0 individuals required in each year between 2010 and 2015 to supply every 1.0 FTE position.

Remote NT communities were also found to have an increased reliance on agency employed nurses since 2004, with use of this comparatively expensive labour force plateauing at a higher level since about 2011. This is in addition to NTG DOH employed agency nurses on casual and temporary contracts. Increased reliance on short-term agency nurses carries further risks of discontinuity of service and diminished health outcomes in a vulnerable Aboriginal population. The risk not only affects the continuity of the health service but also affects continuity of care, which is important in a setting in which interpersonal relationships are fundamental to providing high quality, culturally safe primary care [9]. The high use of agency employed nursing staff has also been identified as increasing risks to nurse safety in remote communities and contributing to ‘orientation burnout’ and higher levels of anxiety amongst permanent staff about their agency nurse colleagues’ skills and knowledge [9].

Our analysis revealed limited evidence of the effectiveness of the NTG DOH programs to strengthen career pathways for nurses and AHPs in remote Aboriginal communities. Level 3 (N3) training positions for remote area nurses increased over the study period, but comprised a supply of less than a headcount of 10 (about 6% of nurse headcount) in total. Given the evidence for high turnover rates for nurses working in remote communities, it is likely that many more nurse training positions in remote communities are required to provide a sustainable supply of level 4 and 5 nurses with the skills necessary to work independently in remote Aboriginal communities. Providing high quality, positive remote training experiences for N3 nurses, however, is critical to their development as remote area nurses. A compounding challenge is that many remote communities lack the stability of supply of experienced, established nurses and AHPs with the capacity to train and support N3 nurses to become the future healthcare workforce.

Our research also suggests that overall supply and career paths for AHPs are problematic, with total headcounts in 2015 for AHPs declining substantially to 61% of what they were in 2010, making 2015 headcounts comparable to those of 2004. The marked decline in AHP supply occurred across levels 1–3. These findings are consistent with a growing body of literature articulating the difficulties faced by Aboriginal people in entering and remaining in the health workforce. These include, for example, difficulties with balancing family and community responsibilities, poor levels of secondary education, and a range of structural and systemic barriers within the AHP health profession [19, 20]. One key systemic barrier identified is the mismatch between the amount of training received and the levels of responsibility and community expectations of AHPs [14]. Another is a lack of opportunities for career progression [14].

This study is not without limitations. First, workforce supply data have been sourced from two distinct datasets, with supply of agency employed nurses unable to be directly assessed, but instead inferred from expenditures on nurse labour hire. Secondly, prior to July 2007 no data on employee age and gender were available, limiting analysis by these variables. Thirdly, some cost centre structures for small clinics have changed due to changes of government and organisational restructuring. Some expenditures on agency nurse labour hire in remote clinics were recorded in a centralised cost centre pool, which meant that they were unable to be allocated to a specific remote clinic.

This study has highlighted important areas for further research. Overall degree of reliance of remote NTG DOH communities on agency nursing staff, while high, is yet to be directly measured and tracked over time. Additionally, studies which use individual-level data to investigate different patterns of use of agency nursing staff by different remote communities are still needed. Our study also indicates that it could be important to differentiate the range of different ways in which nurses are employed, so that we can develop a more sophisticated understanding of how employment structures relate to changes in remote workforce supply and turnover. Finally, the important professional support provided to nurses and AHPs via intermittent visits and telephone support from medical officers requires further research. The role and importance of professional support provided to resident staff by a range of visiting allied health and specialist doctors also requires further investigation.

Despite acknowledged limitations and many remaining evidence gaps, this research nevertheless provides important evidence of long-term trends in supply and sustainability of the resident primary care practitioner workforce in remote NT communities. This evidence is crucial information for policy makers and funders when planning to improve recruitment and retention in remote communities.

## Conclusions

Despite substantial funding boosts from 2007, some funds were expended on staff in regional centres and only a modest increase in nurse and AHP FTE supply was evident in remote communities, with most remote health clinics unable to sustain initial increases in NTG employed nurses and AHPs. Instead, a heavy reliance on short-term agency employed nurses and high turnover of NTG employed staff was evident. Recent declines in AHP supply and low numbers of nurses in remote practice training suggest that the imperative remains to invest in developing stronger career pathways for AHPs and nurses and implementing more robust health service models which better support the supply and retention of long term clinical staff.

## Abbreviations

AHPs: Aboriginal Health Practitioners
DOH: Department of Health
FTE: Full time equivalent
GAS: Government Accounting System
NT: Northern Territory
NTER: Northern Territory Emergency Response
NTG: Northern Territory Government
PIPS: Personnel Information and Payroll Systems
